# Dynamic Model of Polished Stone Value Attenuation in Coarse Aggregate

**DOI:** 10.3390/ma13081875

**Published:** 2020-04-16

**Authors:** Jingyi Liu, Bowen Guan, Huaxin Chen, Kaiping Liu, Rui Xiong, Chao Xie

**Affiliations:** 1 School of Materials Science and Engineering, Chang’an University, Xi’an 710064, China; bguan@chd.edu.cn (B.G.); hxchen@chd.edu.cn (H.C.); zykpliu@chd.edu.cn (K.L.); xiongrui@chd.edu.cn (R.X.); 2015221178@chd.edu.cn (C.X.); 2 The Traffic Paving Materials Engineering Research Center of ministry of education of China, Xi’an 710064, China

**Keywords:** road engineering, coarse aggregate, polished stone value, dynamic model, fractal box dimension, Vickers hardness

## Abstract

The polished stone value (PSV) of coarse aggregate is closely related to pavement skid resistance and traffic safety. However, the determination of the PSV of coarse aggregate is conventionally a time- and energy-intensive process. To facilitate the test process of PSV in materials selection and pavement design and for the prediction of the service life of aggregate materials in practical service, here a new mathematical model of PSV attenuation in coarse aggregate, which employs a physical polishing process analysis, is proposed. The PSVs of four types of coarse aggregates (calcined bauxite, granite, basalt, and limestone) were analyzed through a polishing experiment, and the corresponding mechanism was investigated via scanning electron microscopy analysis. The modeling results are in good agreement with experimental results. The aggregate PSV is affected by both the macrotexture and microtexture of the aggregate surface. The PSV due to the macrotexture exhibits a strong negative correlation with the Vickers hardness of the aggregates and decreases exponentially as the polishing time increases. The attenuation rate decreases as the fractal box dimension in the aggregate surface morphology increases. The primary factor influencing the macrotexture service life and the half-life is the aggregate surface morphology. The PSV due to the microtexture exhibits a strong positive correlation with the Vickers hardness of the aggregates, whereas there is a poor correlation with the aggregate surface morphology and polishing time. The proportion of the aggregate PSV due to the microtexture increases as the aggregate hardness increases. These results highlight the effectiveness of a new modeling approach that may potentially assist in predicting the anti-slip performance and durability of coarse aggregates.

## 1. Introduction

Skid-resistance of a pavement is considered one of the most important factors affecting traffic safety [1], as good anti-skid performance can reduce traffic accidents by providing sufficient adhesion for the vehicle tires, while low skid resistance means poor friction and can increase the risk of accidents. The exposed aggregate on the pavement surface will gradually become polished and worn by traffic, which will reduce its anti-skid performance and eventually create a safety hazard. Investigations show that many highway traffic accidents are related to insufficient pavement skid resistance [2,3], with the particle shape, physical properties, and variations in the coarse aggregate on the pavement surface exerting important effects on its anti-skid performance [4,5].

The polished stone value (PSV) of coarse aggregate is an indicator that reflects the ability of coarse aggregate to resist the polishing action of tires and is used to characterize the ability of coarse aggregate to maintain a certain coefficient of friction against tire abrasion. The PSV of coarse aggregate is closely related to pavement skid resistance. Pavement surfaces with a larger PSV possess a higher friction coefficient. Pavement surfaces with high PSV coarse aggregate have a higher skid resistance, which improves vehicle safety [6,7,8]. Therefore, the relevant technical specifications of asphalt pavements in China have clear requirements and provisions on the PSV of coarse aggregate, which is a key index for determining whether a certain aggregate can be used in the anti-sliding abrasion layer of an asphalt pavement. However, determining the PSV of coarse aggregate is a time- and energy-intensive process. It is therefore of great significance to study the skid resistance characteristics of coarse aggregate and derive a PSV attenuation law to facilitate better-informed material selection for pavement design and construction and also predict the service life of aggregate materials in practical settings.

Numerous research efforts have been reported on the pavement skid resistance problem; these studies investigated the influence of aggregate type [9,10,11], mineral composition [12], particle gradation [13,14], and surface morphology [15,16] on pavement skid resistance. While important advances have been made through these studies, the dynamics of PSV attenuation in coarse aggregate have rarely been reported.

Here a mathematical PSV attenuation model for coarse aggregate was constructed through a theoretical analysis of the polishing process to investigate the sliding-resistance changes in coarse aggregates over time, and experimental observations were analyzed and compared to validate the proposed model, for simplifying the PSV test process and predicting the performance of the coarse aggregates in service.

## 2. Establishment of the PSV Attenuation Dynamic Model for Coarse Aggregate

### 2.1. Polishing Process Analysis

The PSV of coarse aggregate is its surface friction coefficient, which is measured using a pendulum friction coefficient meter after polishing the coarse aggregate with an accelerated polishing machine and a silicon carbide agent. PSV effectively characterizes both the frictional properties and abrasion resistance of the aggregate.

The surface roughness of coarse aggregate has an important effect on its slip resistance. A rougher surface generally has a better slip resistance and higher corresponding PSV than a smoother surface. However, different rocks with the same roughness often have different slip resistances owing to their varying material properties such as mineral type and particle size and distribution. The slip resistance can also be different in the same rock type due to differences in its mineral composition, crystal size, and weathering degree. Therefore, the PSV of aggregate is related to both the surface roughness and material properties of the aggregate.

The surface roughness of coarse aggregate includes both its macrotexture and microtexture [17]. The macrotexture morphology of coarse aggregate surface forms during the aggregate formation process. This macrotexture morphology is directly in contact with the wheels and is gradually polished off since the aggregate surface is exposed on the pavement surface in actual road applications. It is therefore more appropriate for the aggregate application environment to characterize the aggregate morphology based on the macrotexture height of the aggregate surface.

Both the macrotexture and microtexture of the aggregate affect its PSV. The general observation is that the surface friction performance is primarily influenced by the macrotexture at the beginning of the coarse aggregate polishing process, whereas the microtexture is the primary contributor during the later stage of polishing [18].

### 2.2. General Hypothesis

Coarse aggregate is macroscopically homogeneous; its physical properties are stable during the polishing process; and its hardness and other physical properties do not change with polishing time.

**Figure 1:**
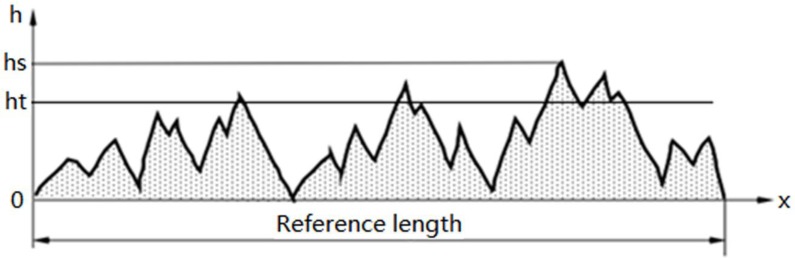
Schematic of the macrotexture morphology of the aggregate surface.

The macrotexture morphology of the original coarse aggregate surface is irregularly distributed like mountain peaks, as shown in Figure 1. The vertical coordinate *h* in the figure represents the macrotexture height of the aggregate; the *x*-coordinate is the length along the aggregate surface, where *h*_s_ is the initial maximum macrotexture height and *h*_t_ is the macrotexture height of the aggregate surface at time *t*.

### 2.3. Macrotexture Analysis and Hypothesis

The macrotexture of the aggregate surface undergoes a process of constant wear and tear during the aggregate polishing experiment, such that the macrotexture height decreases with increasing polishing cycles. As the number of polishing cycles is a function of the polishing speed and polishing time, the macrotexture height of the aggregate surface is a function of polishing time for a given polishing speed. This means that the macrotexture height decreases with increasing polishing time. The macrotexture height of the aggregate surface is lowered as the polishing time increases until the macrotexture height approaches zero.

The wear area increases and the corresponding wear resistance increases as the macrotexture height decreases during the polishing process since the macrotexture is a distribution of peaks (Figure 1). This results in a larger area that is subject to wear, greater energy requirements for additional wear, greater abrasion resistance, and a slowdown in the reduction rate of the macrotexture height. Conversely, a higher macrotexture possesses a smaller worn peak area, requires less energy for additional wear, is easier to wear, possesses a smaller wear resistance, and has a higher macrotexture height attenuation rate. This indicates that the macrotexture height attenuation rate is proportional to the macrotexture height.

Furthermore, fractal theory can be used since the aggregate surface roughness has statistical self-similarity [19]. Fractal theory is a methodology to measure or describe the complexity of complex systems. Fractal geometry represents the research object with an irregular geometry. The fractal dimension is one of the main parameters in fractal theory, which is a measure of the irregularity of a complex shape. The fractal dimension has many definitions and calculation methods such as the Hausdorff dimension, similarity dimension, information dimension, counting box dimension, and correlation dimension. Here the box-counting dimension, also known as the MinKeFu kiwi number, is measured and calculated using a box of different scales on the surface of the object, which provides a quantitative description of the complexity of the fractal collection. We employ the box dimension to express the complexity of the aggregate surface morphology. The fractal dimension abrasion resistance increases as the surface morphology increases, where the macrotexture height attenuation rate decreases, such that the macrotexture height attenuation rate is inversely proportional to the fractal box dimension of the aggregate surface:

d*h*_t_/d*t* ∝ −*h*_t_/*D* (1)

where *h*_s_ and *h*_t_ are the initial aggregate macrotexture height and macrotexture height after polishing time *t*, as shown in Figure 1, and *D* is the fractal box dimension of the initial aggregate surface.

The negative sign in Equation (1) means that the rate of change in the macrotexture height exhibits a decreasing trend. Therefore, Equation (1) can be rewritten as

d*h*_t_/d*t* = −*kh*_t_/*D* (2)

where *k* (s^−1^ or min^−1^) is a constant related to the equipment conditions during aggregate wear.

We then integrate Equation (2) to obtain

*h*_t_ = *h*_s_e^−^*^kt/D^* (3)

As the polished value owing to the macrotexture corresponds to the macrotexture height, the polished value at time *t (P_t_)* owing to the macrotexture can be defined as

*P*_t_ = *P*_s_e^−^*^kt/D^* (4)

where *P*_t_ is the polished value at time *t* and *P*_s_ is the polished value for *h*_s_. As *P*_t_ is related to both the surface morphology and material properties of the aggregate, the exponential and *P*_s_ in Equation (4) capture the influence of the surface morphology and material properties of the aggregate, respectively. Given that e^−^*^kt/D^* is a number between 0 and 1, *P*_t_ tends to 0 with increasing polishing time, with *P*_s_ representing the maximum macrotexture polished value during the polishing process.

### 2.4. Microtexture Analysis and Hypothesis

The microtexture of coarse aggregate presents a different situation during the polishing process. The polished value owing to the microtexture of the aggregate is influenced by the mineral composition and properties, grain size, defect size, and their respective distributions during the polishing process since the rock is formed of crystals with different strengths, grain sizes, and defects [20], this highlights that the aggregate hardness is one of the primary factors that affects its polished value [21].

The soft grains and defects in high-hardness aggregates are constantly worn down during the polishing process, whereas the hard grains are more difficult to grind and are the main components that hinder abrasion and friction. The hard particle bulges will gradually form a smooth macrostructure area, but as an uneven microstructure area, as the polishing process progresses. The smooth macrostructure area formed by abrasion decreases as the aggregate hardness increases. Therefore, the smooth macrostructure area that is formed by hard grains is the primary surface morphology feature during the polishing process.

Conversely, the grains in low-hardness aggregates are easier to grind down because the grains are softer. The abrasive will usually form abrasive wear on the aggregate surface if the aggregate hardness is much lower than the particle hardness of the abrasive. Therefore, the continuously grinding down of the grains and the formation of wear furrows on the surface of soft aggregates are the main surface morphology characteristics during the polishing process.

A microtexture will always exist during the aggregate polishing process, regardless of whether the aggregate is hard or soft. A new surface is constantly forming along the aggregate surface, such that its friction capacity is unaffected, even as the height of the aggregate surface continues to decrease. Therefore, the polished value owing to the microtexture is largely unrelated to the microtexture height of the aggregate surface and polishing time.

However, the friction effect on the abrasives is different owing to the different morphologies of the new surface, which are formed by the hard and soft aggregates. Therefore, the PSV due to the microtexture is related to the aggregate material hardness.

Hardness is an important performance index for measuring the degree of hardness or softness in materials. It is a comprehensive index of mechanical properties of the material, such as elasticity, plasticity, strength, and toughness. Studies have shown that the hardness of the material itself is related to the chemical bonds of the crystal. The aggregate material with higher hardness is the harder material and exhibits greater resistance to friction between the abrasives during the aggregate polishing process. Conversely, the aggregate material with lower hardness is the softer material and exhibits a weakened ability to prevent friction and wear of the abrasives. Therefore, the polished value owing to the microtexture is proportional to the aggregate hardness:

*P*_x_ ∝ *H*_v_ (5)

where *P*_x_ is the polished value owing to the microtexture and *H*_v_ is the Vickers hardness of the aggregate. This can be rewritten as

*P*_x_ = *b*_0_ + *b*_1_*H*_v_ (6)

where *b*_0_ and *b*_1_ are adjustment coefficients related to the test environment and polishing conditions.

### 2.5. Assumption of the PSV Attenuation Law for Coarse Aggregate during Polishing

PSV comprises the macrotexture and microtexture influences as follows:

PSV = *P*_t_ + *P*_x_ = *P*_s_e^−^*^kt/D^* + *P*_x_ (7)

The initial total polished value simplifies to *P*_0_ = *P*_s_ + *P*_x_ when *t* = 0, such that the relationship between the initial total polished value *P*_0_ and maximum *P*_s_ value is

*P*_s_ = *P*_0_ − *P*_x_ (8)

The substitution of Equation (8) into Equation (7) yields:

PSV = (*P*_0_ − *P*_x_)e^−^*^kt/D^* + *P*_x_ (9)

*P*_0_ is the total friction coefficient of the aggregate at *t* = 0 and the maximum PSV during aggregate polishing. *P*_0_ is also related to the aggregate material properties (hardness) since it is the sum of *P*_s_ and *P*_x_, both of which are related to the aggregate material properties (hardness).

*P*_0_ is proportional to the aggregate hardness:

*P*_0_ ∝ *H*_v_ (10)

which can be rewritten as,

*P*_0_ = *a*_0_ + *a*_1_*H*_v_ (11)

where *a*_0_ and *a*_1_ are adjustment factors.

The substitution of Equations (6) and (11) into Equation (9) yields

PSV = [(*a*_0_ − *b*_0_) + (*a*_1_ − *b*_1_)*H*_v_] e^−^*^kt/D^* + *b*_0_ + *b*_1_*H*_v_ (12)

Equation (12) is the mathematical model of aggregate PSV as a function of time. This tells us that the aggregate PSV is affected by both the macrotexture and microtexture, such that its value is related to *H*_v_ and *D* and decreases exponentially with polishing time. *k*, *a*_0_, *a*_1_, *b*_0_, and *b*_1_ are constants for a given test equipment condition. These constants can be obtained by measuring the *P*_0_, *H*_v_, and *D* values of the coarse aggregates and PSV values of the partial polishing time, which then allows the aggregate PSV attenuation over time to be obtained.

## 3. Model Validation

### 3.1. Test Materials

The experimental materials used for the model verification are the same as those in the literature [22], with calcined bauxite, granite, basalt, and limestone chosen as the test aggregates. The basic physical and mechanical properties of these aggregates are listed in Table 1.

### 3.2. Experimental Methods

PSV test data from the literature [22] were used in the model, where the rotation of the polishing machine was 320 ± 5 rpm. The polishing time was obtained by dividing the number of polishing cycles by the rotational speed of the polishing machine.

A VK-9700 color 3D laser microscope (Keyence; Osaka, Japan) and S-4800 scanning electron microscope (Hitachi; Tokyo, Japan) were used to observe the aggregate morphology during polishing.

### 3.3. Determination of the Model Parameters

#### 3.3.1. Determination of the Fractal Box Dimension *D* of the Aggregates

**Figure 2:**
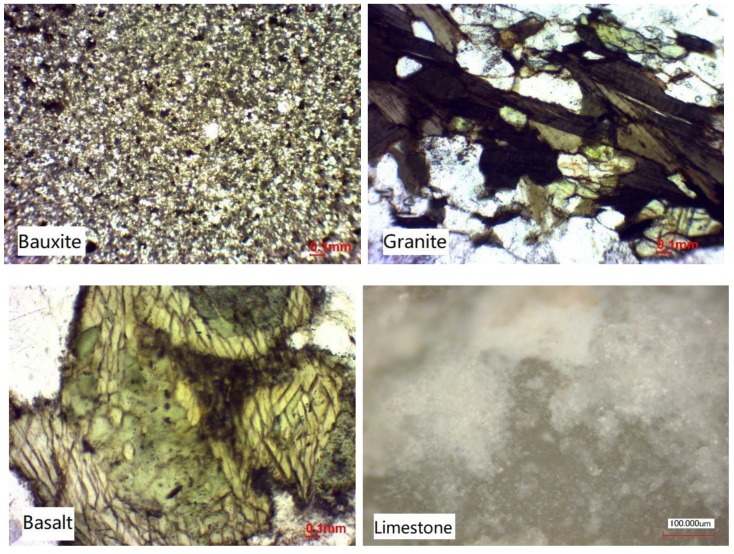
Laser microscope images of the four aggregates.

**Figure 3:**
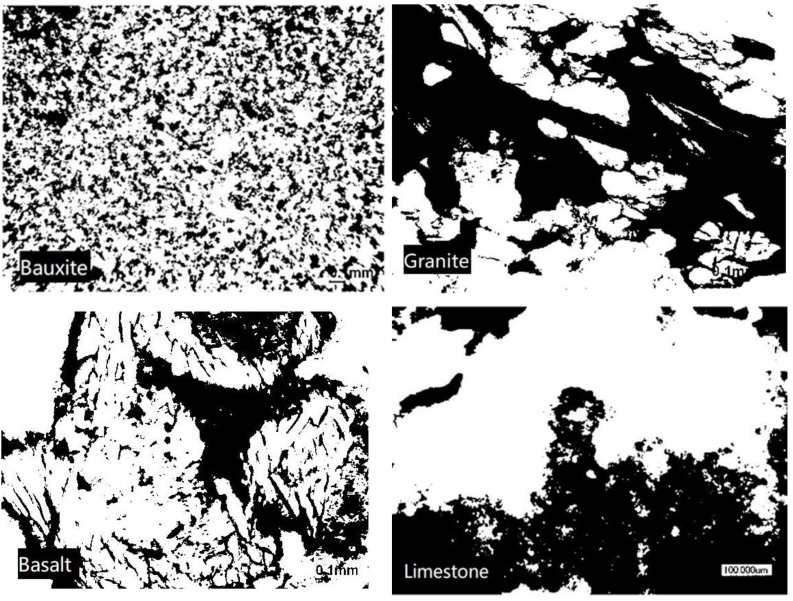
Binarization images of the four aggregates, derived from the laser microscope images in Figure 2.

Laser microscope images of the sections from the aggregate samples used in the experiment are shown in Figure 2. MATLAB (R2017b, MathWorks, Natick, MA, USA) was used to binarize the photos in Figure 2; then the square box was taken as the counting cell to calculate the fractal box dimension. The binarization image of each laser microscope image is shown in Figure 3. The fractal box dimensions of the four aggregates are listed in Table 2.

#### 3.3.2. Determination of the *k* Value

Some of the experimental polished values for limestone [22] were selected to determine the *k* value, which are listed in Table 3. Table 3 indicates that *P*_0_ = 67.7. We assume that *t*→∞ at *t* ≈ 52,500 min, such that *P*_∞_ = *P*_x_ ≈ 29.7. The *k* value is then derived from Equation (9), where

*k* = −*D*/*t* × ln((PSV − *P*_x_)/(*P*_0_ − *P*_x_)) (13)

PSV = 38.4 at *t* = 15,000 min (Table 3), and *D* = 1.9562 for limestone (Table 2), yielding

*k* = 0.000192 min^−1^

#### 3.3.3. Determination of the *P*_x_ Values

The PSVs of the four aggregate samples at *t* = 0 and 15,000 min were selected to determine the *P*_x_ values, with the analyzed experimental values [22] listed in Table 4. The *P*_x_ values for the four aggregates were then determined by rearranging Equation (9):

*P*_x_ = (PSV − *P*_0_e^−*kt*/*D*^)/(1 − e^−*kt*/*D*^) (14)

with the determined *k* value, PSV values at *t* = 0 and 15,000 min, and *D* values of the four aggregates used to calculate the *P*_x_ values, which are listed in Table 5.

#### 3.3.4. Determination of the *b*_0_, *b*_1_, *a*_0_, and *a*_1_ Values

The *H*_v_ values in Table 1, *P*_x_ values in Table 5, and Equation (6) were used to determine the *b*_0_ and *b*_1_ values, with a regression analysis yielding

*b*_0_ = 27.15472 and *b*_1_ = 0.015138

The correlation coefficient *R* between *P*_x_ and *H*_v_ is 0.956 at the 95% confidence interval, indicating that *P*_x_ exhibits a strong linear correlation with *H*_v_, where:

*P*_x_ = 27.15472 + 0.015138*H*_v_ (15)

The *H*_v_ values in Table 1, *P*_0_ values in Table 4, and Equation (11) were used to determine the *a*_0_ and *a*_1_ values, with a regression analysis yielding

*a*_0_ = 63.39083 and *a*_1_ = 0.007985

The correlation coefficient *R* between *P*_0_ and *H*_v_ is 0.981 at the 95% confidence interval, indicating that *P*_0_ exhibits a strong linear correlation with *H*_v_, where:

*P*_0_ = 63.39083 + 0.007985*H*_v_ (16)

The substitution of Equations (15) and (16) into Equation (12) yields the total PSV:

PSV = (36.236 − 0.00715*H*_v_) e^−0.000192*t*/*D*^ + 27.155 + 0.01514*H*_v_ (17)

### 3.4. PSV Validation

**Figure 4:**
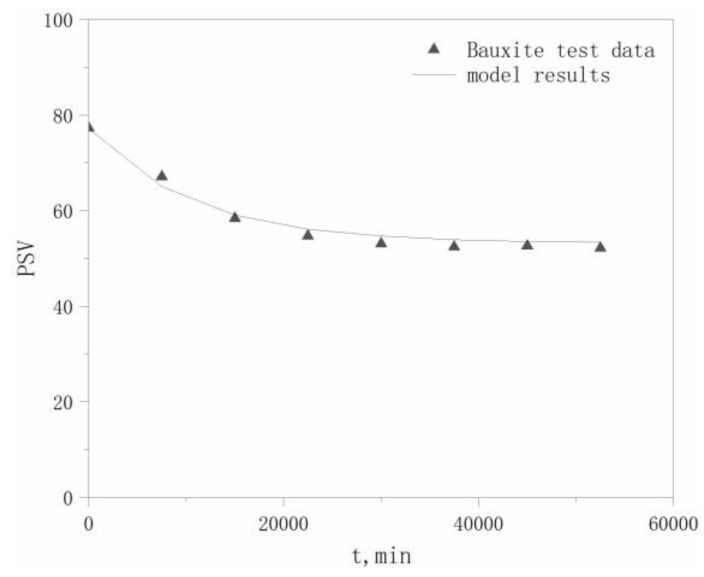
Modeled and tested PSV Results for calcined bauxite.

**Figure 5:**
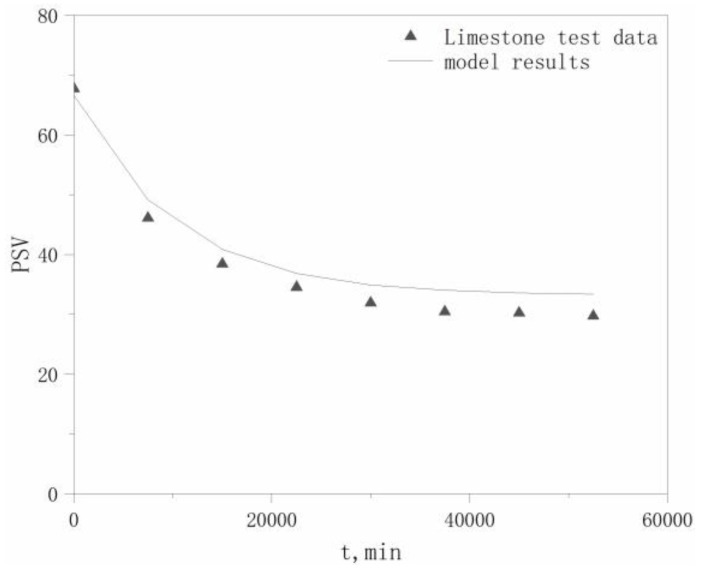
Modeled and tested PSV results for granite.

**Figure 6:**
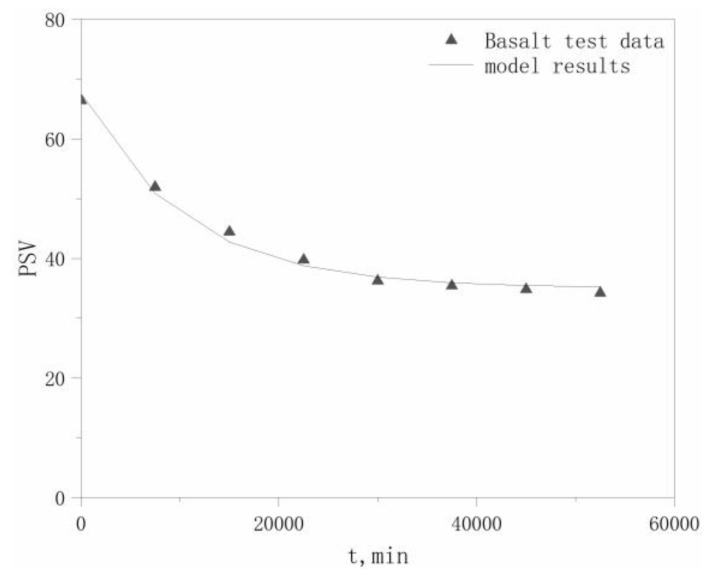
Modeled and tested PSV results for basalt.

**Figure 7:**
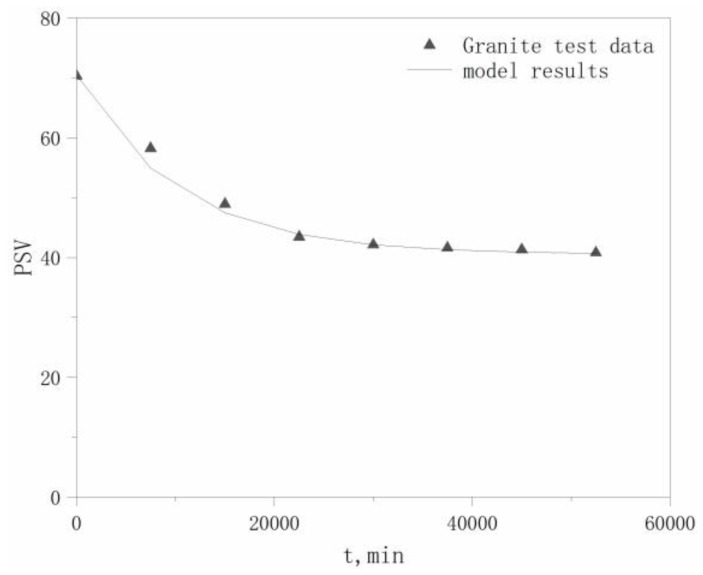
Modeled and tested PSV results for limestone.

PSV test data for the four aggregates in the literature [22] were selected to validate the model, which are listed in Table 6. The PSVs of the aggregates at different polishing times *t* are calculated using Equation (17), with comparisons between the modeled and experimental values for the four aggregates shown in Figure 4, Figure 5, Figure 6 and Figure 7.

The maximum relative deviation between the modeled and experimental PSVs, relative deviation of the maximum *P*_0_ values, and relative deviation of the *P*_x_ values, which are derived from Figure 4, Figure 5, Figure 6 and Figure 7, are listed in Table 7. The accuracy of the modeled PSV results, ranging from high to low, is calcined bauxite > basalt > granite > limestone. The relative PSV deviation due to the microtexture, ranging from high to low, is limestone > basalt > calcined bauxite > granite.

The results in Table 7 show that the differences between the modeled and experimental results are not large, with a maximum deviation of only 12.3%, which indicates that our hypothesis is reasonable. Therefore, our proposed model can adequately describe the PSV attenuation law for coarse aggregates.

## 4. Analysis of the PSV Decay Law

### 4.1. Microtexture Polished Values (P_x_)

#### 4.1.1. Factors Influencing the *P*_x_ Values

**Figure 8:**
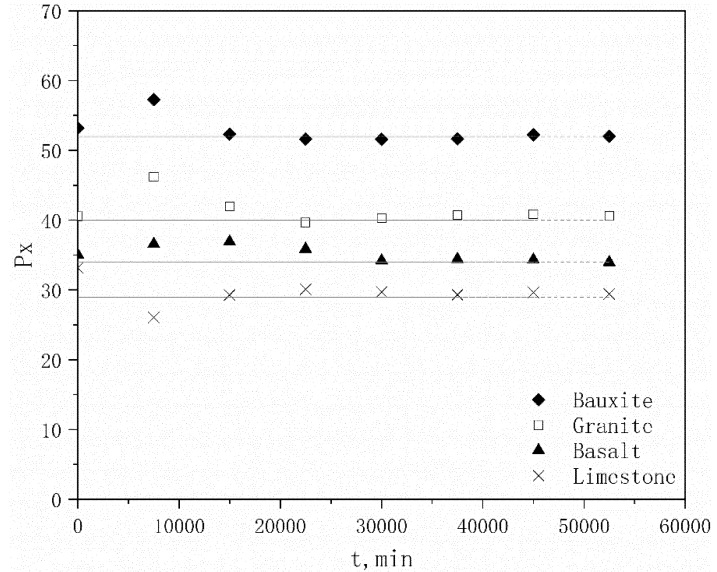
*P*_x_ values as a function of time *t* for each aggregate.

The *P*_x_ values for the four aggregates at different times *t* are shown in Figure 8.

The *P*_x_ values for each aggregate are generally constant over time, with the exception of some individual points, which indicates that the *P*_x_ value is not a function of polishing time, such that any changes in *P*_x_ are likely unrelated to the polishing time. However, there are obvious differences among the aggregates, which means that the material properties have a major influence on *P*_x_. The average *P*_x_ value of each aggregate is listed in Table 8.

The correlation coefficient *R* between the average *P*_x_ value and *H*_v_ for each aggregate is 0.984 at the 95% confidence interval, which indicates that *P*_x_ does have a strong linear correlation with the aggregate hardness [23], such that the *P*_x_ value will increase as the aggregate hardness increases.

#### 4.1.2. *P*_x_/*P*_s_ Ratio

The influence of the microtexture is analyzed by comparing *P*_x_ with *P*_s_; the results are listed in Table 9. The *P*_x_/*P*_s_ ratios of the aggregates exhibit the following trend (ranging from high to low): calcined bauxite > granite > basalt > limestone (Table 9). The first three values are much higher than the last value. As *P*_0_, which is the maximum PSV, is composed of *P*_x_ and *P*_s_, the high *P*_x_/*P*_s_ ratios indicate that a high proportion of *P*_x_ values is derived from the high-hardness aggregate, whereas a low proportion of *P*_s_ values is derived from the low-hardness aggregate, and vice versa.

The correlation coefficient *R* between the *P*_x_/*P*_s_ ratio and *H*_v_ for the four aggregates is 0.985 at the 95% confidence interval, demonstrating a strong linear correlation between the *P*_x_/*P*_s_ ratio and *H*_v_. This means that the *P*_x_/*P*_s_ ratio increases as the hardness of the aggregate material increases, which indicates that the microstructure exerts a greater influence on PSV. Here the *P*_x_ value for calcined bauxite, which possesses high hardness, accounted for more than 2/3 of the *P*_0_ value, whereas the *P*_x_ value of limestone, which possesses low hardness, accounted for less than 1/2 of the *P*_0_ value. Therefore, the PSV of the high-hardness aggregates is primarily controlled by *P*_x_, whereas the PSV of the low-hardness aggregates is affected by both *P*_x_ and *P*_t_ [24].

### 4.2. Macrotexture Polished Values P_s_

#### 4.2.1. Changes in Maximum *P*_s_

The *P*_s_ values for each aggregate are calculated using Equation (8) and are listed in Table 10. The *P*_s_ values of the high-hardness aggregates (calcined bauxite and granite) are small, whereas the *P*_s_ values of the low-hardness aggregates (basalt and limestone) are large. The correlation coefficient between *P*_s_ and *H*_v_ for the four aggregates is −0.883, which indicates that there is a strong negative correlation between *P*_s_ and *H*_v_.

Equations (15) and (16) show that *P*_s_ = (*a*_0_ − *b*_0_) + (*a*_1_ − *b*_1_)*H*_v_ = 36.236 − 0.00715*H*_v_, where the negative change in slope means that *P*_s_ decreases as the aggregate hardness increases. This is consistent with the calculated results in Table 10, which indicates that the macrotexture is preserved, the height value is high, the corresponding aggregate surface macroscopic peak friction and wear area are small, and the friction and wear resistance are small for high-hardness aggregates during polishing, such that their *P*_s_ values are also small. The opposite trend occurs for low-hardness aggregates.

#### 4.2.2. Macrotexture Service Life *t*_0.05_ and Half-Life *t*_0.5_

Equation (4) highlights that the *P*_t_ value of the macrotexture decreases exponentially with increasing polishing time *t*, and is related to the maximum *P*_s_ and *D*. Note that the *P*_t_ decay rate will decrease as *D* increases.

The macrotexture reaches the maximum service life during the aggregate polishing process when the *P*_t_ value owing to the macrotexture height approaches zero. However, a long polishing time is required for the *P*_t_ value to approach zero in practical engineering. Therefore, we assume that the *P*_t_ service life has been reached when *P*_t_ = 0.05*P*_s_. Equation (4) is simplified to e^−^*^kt/D^* = 0.05, and solved as follows:

*t*_0.05_ = −*D*/*k* × ln(0.05) = 2.99573 × *D*/*k* (18)

where *t*_0.05_ is the service life.

Similarly, we assume that the *P*_t_ half-life has been reached when *P*_t_ = 0.50*P*_s_, such that

*t*_0.5_ = −*D*/*k* × ln(0.5) = 0.69315 × *D*/*k* (19)

where *t*_0.5_ is the half-life. The *t*_0.05_ and *t*_0.5_ values for the four aggregates were calculated using Equations (18) and (19), respectively, and are listed in Table 11.

The *t*_0.05_ and *t*_0.5_ values for the four aggregates follow the same decreasing trend, where calcined bauxite > basalt > granite > limestone, but they do exhibit small differences. The maximum deviation occurred between calcined bauxite and limestone, although the value did not exceed 4.6%. However, there is a >3-fold difference in *H*_v_ between these two aggregates, which indicates that the aggregate material properties have little influence on the *t*_0.05_ and *t*_0.5_ of the macrotexture. The main factor influencing the *t*_0.05_ and *t*_0.5_ of the *P*_t_ value should be the aggregate surface morphology, which is represented by the *D* value of the aggregate surface in this paper. Both *t*_0.05_ and *t*_0.5_ are linearly related to *D*, as shown in Equations (18) and (19), which indicates that both *t*_0.05_ and *t*_0.5_ increase as the aggregate surface morphology becomes more complex. Furthermore, the slope of *t*_0.05_ is more sensitive to changes in *D* than that of *t*_0.5_, which reveals that increasing the box dimension (increasing the complexity of the aggregate surface morphology, such as increasing its angularity) is an effective way to extend the service life of the aggregate macrotexture [25].

### 4.3. Influence of the Material Property Difference on the PSV Mechanism

**Figure 9:**
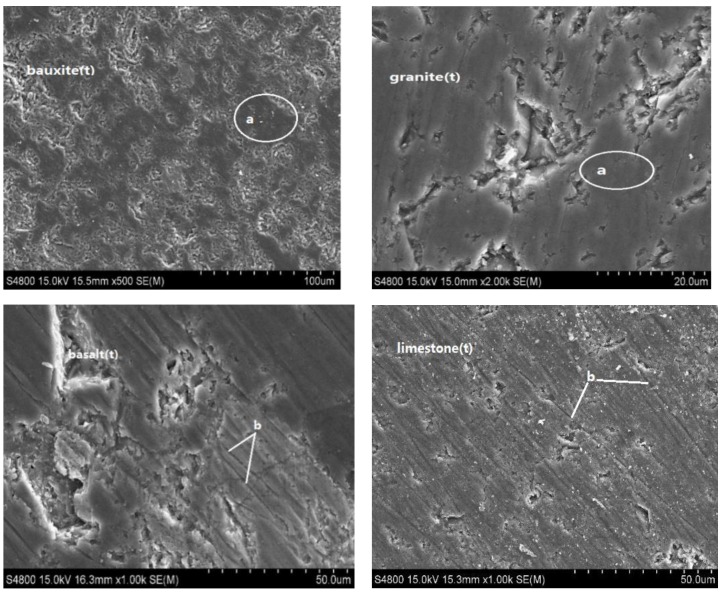
SEM images of the four polished aggregates. a: hard particles that have been polished to a smooth point; b: furrows.

Scanning electron microscopy (SEM) microstructure analysis was conducted on four types of aggregates to investigate how the aggregate material properties influence the polished value, with SEM photos at *t* = 37,500 min (200,000 cycles of polishing) shown in Figure 9. The aggregate surface morphologies exhibit key differences after polishing.

Obvious smooth macrostructure areas and uneven microstructure points (point a in Figure 9) formed on the surface of the hard particles in the high-hardness aggregates (calcined bauxite and granite) during polishing, which are the primary structures that inhibit abrasion and further surface wear, such that *h*_s_ and *P*_s_ are lower. The hardness of granite is lower than that of bauxite, so its smooth macrostructure area is clearly larger than that of bauxite, which is consistent with the previous analysis results. Conversely, the surfaces of the low-hardness aggregates (basalt and limestone) were obviously worn after polishing, such that abrasion could not be effectively inhibited, yielding higher *h*_s_ and *P*_s_ values.

The smooth points that formed on the bauxite and granite surfaces also exhibited a strong resistance to friction during polishing, indicating a high *P*_x_ value. However, the abrasives in basalt and limestone adopted high-hardness emery (SiC) particles during the polishing experiments, with the emery particles forming obvious wear furrow scratches on the soft aggregate surface (point b in Figure 9). These soft aggregates are easy to wear, such that their resistance to the frictional movement of the abrasives is low, indicating a low *P*_x_ value. Regardless, the *P*_x_ value of the aggregates does not change with polishing time due to a new worn surface being formed repeatedly during the polishing process, such that *P*_x_ is only related to the aggregate material.

## 5. Conclusions

(1) Through a physical polishing process analysis, a new mathematical model of PSV attenuation in coarse aggregate is proposed. In the model, the PSV of coarse aggregate is affected by both the macrotexture and microtexture. Its value is related to the material properties, aggregate surface morphology, and polishing time. The calculated data of the proposed PSV model are in good agreement with the experimental results.

(2) The polished value owing to the macrotexture is negatively correlated with the coarse aggregate hardness and decreases exponentially as the polishing time increases. The decay rate decreases as the fractal box dimension of the aggregate surface morphology increases. The primary factor influencing the service life and half-life of the macrotexture is the aggregate surface morphology. Both the service life and half-life increase as the fractal box dimension increases.

(3) The polished value owing to the microtexture is positively correlated with the Vickers hardness of coarse aggregate and is less correlated with the surface morphology and polishing time. The proportion of the PSV that consists of the polished value owing to the microtexture increases with increasing aggregate hardness.

## Figures and Tables

**Table 1:** Properties of the test aggregates.

Physical Index	Calcined Bauxite	Granite	Basalt	Limestone
Apparent density, g cm^−3^	3.228	3.035	2.826	2.722
Crushed stone value, %	7.74	11.5	12.5	22.6
Los Angeles test (LAA), %	10.6	12.9	21.1	20.6
Vickers hardness, *H*_v_	1721.33	884.85	520.46	397.07

**Table 2:** Fractal box dimension *D* of the four aggregates.

	Calcined Bauxite	Granite	Basalt	Limestone
Fractal box dimension (*D*)	2.0454	1.9699	2.002	1.9562

**Table 3:** Limestone polishing test data used to determine the *k* value [22]. PSV, polished stone value.

t, min	0	15,000	52,500
PSV	67.7	38.4	29.7

**Table 4:** Experimental PSV test results for the four aggregates (from [22]).

	Calcined Bauxite	Granite	Basalt	Limestone
*P*_0_ (*t* = 0)	77.3	70.3	66.4	67.7
PSV (*t* = 15,000 min)	58.4	48.9	44.4	38.4

**Table 5:** Calculated *P*_x_ values for the four aggregates.

	Calcined Bauxite	Granite	Basalt	Limestone
Calculated results, *P*_x_	52.28	42.44	37.56	29.68

**Table 6:** PSV test results used to validate the model.

*t*, min	0	7500	15,000	22,500	30,000	37,500	45,000	52,500
Calcined bauxite	77.3	67.1	58.4	54.7	53.1	52.4	52.6	52.2
Granite	70.3	58.2	48.9	43.4	42.1	41.6	41.3	40.8
Basalt	66.4	51.9	44.4	39.7	36.2	35.4	34.8	34.2
Limestone	67.7	46.1	38.4	34.5	31.9	30.4	30.2	29.7

**Table 7:** Relative deviations between the modeled and experimental values for the different aggregates.

	Maximum Relative Deviation of PSV, %	Relative Deviation of *P*_0_, %	Relative Deviation of *P*_x_, %
Calcined bauxite	3.0	0.2	2.3
Granite	6.3	0.2	0.3
Basalt	4.3	1.7	3.0
Limestone	12.3	1.7	12.3

**Table 8:** Average *P*_x_ values for the different aggregates.

	Calcined Bauxite	Granite	Basalt	Limestone
Average *P*_x_	52.74	41.35	35.16	29.59

**Table 9:** *P*_x_/*P*_s_ ratios for the different aggregates.

Aggregate	Calcined Bauxite	Granite	Basalt	Limestone
*P*_x_/*P*_s_	2.15	1.43	1.13	0.78

**Table 10:** Calculated maximum macrotexture polished values *P*_s_ for the different aggregate materials.

Aggregate	Calcined Bauxite	Granite	Basalt	Limestone
*P*_s_	24.56	28.95	31.24	38.11

**Table 11:** Service life *t*_0.05_ and half-life *t*_0.5_ for the four aggregates.

Aggregate	Calcined Bauxite	Granite	Basalt	Limestone
*t*_0.05_, min	31,914	30,736	31,237	30,522
*t*_0.5_, min	7384	7112	7228	7062

## Abbreviation

PSV	polished stone value
SEM	scanning electron microscopy
LAA	Los Angeles test
*h*	macrotexture height of the aggregate
*h*_s_	initial maximum macrotexture height
*h*_t_	the macrotexture height of the aggregate surface at time *t*
*t*	polishing time
*D*	fractal box dimension of the initial aggregate surface
*K*	a constant related to the equipment conditions during aggregate wear
*P*_t_	polished value at time *t*
*P*_s_	polished value for *h*_s_
*P*_0_	total friction coefficient of the aggregate at t = 0
*P*_x_	polished value owing to the microtexture
*P*_∞_	polished value when time t→∞
*H*_v_	Vickers hardness of the aggregate
*b*_0_, *b*_1_	adjustment coefficients related to the test environment and polishing conditions
*a*_0_, *a*_1_	adjustment factors
*R*	correlation coefficient
*t*_0.05_	macrotexture service life
*t*_0.5_	macrotexture service half life
